# Cerebral Tissue Oxidative Ischemia-Reperfusion Injury in Connection with Experimental Cardiac Arrest and Cardiopulmonary Resuscitation: Effect of Mild Hypothermia and Methylene Blue

**DOI:** 10.1007/s12035-017-0723-z

**Published:** 2017-09-11

**Authors:** Lars Wiklund, Ranjana Patnaik, Aruna Sharma, Adriana Miclescu, Hari S. Sharma

**Affiliations:** 1 Laboratory of Cerebrovascular Research, Department of Surgical Sciences, Anesthesiology & Intensive Care Medicine, Uppsala University, S-75185 Uppsala, Sweden; 2 Department of Surgical Sciences, Anaesthesiology & Intensive Care Medicine, University Hospital, Uppsala University, SE-75185 Uppsala, Sweden; 3 National Institute of Technology, School of Biomedical Engineering, Banaras Hindu University, Varanasi, 221005 India

**Keywords:** Cardiac arrest, Oxidative injury, Ischemia reperfusion, Methylene blue

## Abstract

The present investigation is an expansion of previous studies which all share a basic experimental protocol of a porcine-induced cardiac arrest (CA) of 12 min followed by 8 min of cardiopulmonary resuscitation (CPR), different experimental treatments (immediate as well as postponed induced mild hypothermia and administration of much or less cool intravenous fluids), and a follow-up period of 3 h after which the animals were sacrificed. Another group of animals was studied according to the same protocol after 12-min CA and “standard CPR.” After death (within 1 min), the brains were harvested and frozen in liquid nitrogen awaiting analysis. Control brains of animals were collected in the same way after short periods of untreated CA (0 min, 5 min, and 15–30 min). Previous studies concerning chiefly neuropathological changes were now expanded with analyses of different tissue indicators (glutathione, luminol, leucigenin, malonialdehyde, and myeloperoxidase) of cerebral oxidative injury. The results indicate that a great part of oxidative injury occurs within the first 5 min after CA. Immediate cooling by administration of much intravenous fluid results in less cerebral oxidative injury compared to less intravenous fluid administration. A 30-min postponement of induction of hypothermia results in a cerebral oxidative injury comparable to that of “standard CPR” or the oxidative injury found after 5 min of untreated CA. Intravenous administration of methylene blue (MB) during and immediately after CPR in combination with postponed cooling resulted in no statistical difference in any of the indicators of oxidative injury, except myeloperoxidase, and glutathione, when this treatment was compared with the negative controls, i.e., animals subjected to anesthesia alone.

## Introduction

Numerous studies of cerebral ischemia and reperfusion after cardiac arrest have indicated that one of the important mechanisms of cerebral injury is to be found in the creation and action of free oxygen and nitrogen radicals. The cerebral injury resulting from the ischemia/reperfusion after 12-min cardiac arrest (CA) and 8-min cardiopulmonary resuscitation (CPR) and has been documented by our group [1–3]. Our own group has so far monitored the oxidative injury by measuring blood-borne metabolites of fat, 8-iso-PGF_2α_ and 15-keto-PGF_2α_ [4, 5]. Thus, we found that mixed and jugular venous blood contains increasing amounts of these compounds during the first 90 min after reestablishment of spontaneous circulation (ROSC) after which they subside [6, 7]. We have also corroborated that administration of methylene blue (MB), a compound which among other properties also is a scavenger, attenuates the blood concentration of 8-iso-PGF_2α_ when administered during CPR [1, 2, 6, 8]. Similarly, Idris et al. (2005) have documented an increase of the same kind of compounds in cerebral tissue after CA and CPR [9]. Against this background, it seemed logical to determine other oxidative indicators of cerebral tissue injury.

As many of our studies have been performed according to the same protocol, it has been possible to analyze cerebral tissue oxidative indicators in deep frozen specimen from several of our already performed experiments where different experimental trial treatments have been studied.

## Methods

All experiments presented below have been approved by the Regional Animal Review Committee according to local and legislative requirements, one exception being the rat control study performed at National Institute of Technology, Varanasi, India, where appropriate local and federal requirements have been met.

In short, a total of 62 piglets of approximately 25 kg b.w. were anesthetized after which a 12-min untreated cardiac arrest was induced by an alternating transthoracic current. Then, 8 min of CPR followed which often resulted in ROSC. Surviving piglets were monitored for 1 or 3 h after which the piglets were sacrificed and the brain collected and immediately, within 1 min after death, deep frozen in liquid nitrogen, and stored in a − 80° C freezer pending analysis. Parietal gray and white matter were included in the tissue samples. Detailed descriptions of the experimental methods have been published previously [1, 2, 10, 11].

In the first set of experiments, porcine cerebral tissue [2] was harvested within either 0–1 min (“negative controls,” *n* = 5), 5 or 15–30 min after untreated CA, denoted as untreated cardiac arrest 5 min (*n* = 3) and untreated cardiac arrest 15–30 min (*n* = 5).

In the second set of experiments, piglets (*n* = 20) were subjected to 12-min untreated CA followed by 8 min of CPR. Immediately after ROSC, the animals were cooled to 34–33 °C by 5 °C intravenous administration of much (Ringer’s acetate 30 mL/kg) or less, also cooled, (hypertonic saline dextran, 3 mL/kg) fluid combined with external cooling with ice packs. Another four piglets were studied after normothermic (37.5–38.5 °C) “standard CPR” where no hypothermic treatment was instituted [11].

In the third set of experiments, the same kind of CA (12 min) and CPR (8 min) was also followed by induction of mild hypothermia (34–33 °C) by administering 5 °C Ringer’s acetate (30 mL/kg) along with surface cooling by ice packs, but now postponed to 30 min after ROSC (*n* = 5). This group of piglets was compared to another group receiving MB (totally 3 mg/kg) during CPR and 60 min thereafter in addition to the postponed hypothermia of 34–33 °C (*n* = 6). These two groups were compared with a group of seven piglets with 12-min CA and 8-min normothermic CPR, designated as “standard CPR.” To this group, we now added four piglets which were sacrificed and the brain harvested 0–1 min after CA, thus added to the group mentioned above designated as “negative controls” [10].

Finally, ten Wistar rats were sacrificed and the cerebral tissue immediately harvested and frozen in liquid nitrogen, indicated by “rat controls.” This group is the normothermic internal controls of the chemical analysis laboratory.

### Chemical Analyses

#### Cerebral Myeloperoxidase Activity

The activities of brain-associated myeloperoxidase assay were carried out according to commercial protocol [12]. The tissue samples (0.2–0.3 g) were homogenized in ten volumes of ice-cold potassium phosphate buffer (PB, 50 mM K_2_HPO_4_, pH 6.0) containing hexadecyltrimethylammonium bromide (HETAB; 0.5%, *w*/*v*) and centrifuged at 41,400 × g (10 min). The pellets were suspended in 50 mM PB containing 0.5% hexadecyltrimethylammonium bromide. After three freeze and thaw cycles, with sonication between cycles, the samples were centrifuged at 41.400 g for 10 min and aliquots (0.3 mL) were added to 2.3 mL of reaction mixture containing 50 mM PB, o-dianisidine, and 20 mM H_2_O_2_ solution [12]. One unit of enzyme activity was defined as the amount of MPO present that caused a change in absorbance measured at 460 nm for 3 min. The MPO activity was expressed as unit per gram tissue.

##### Malondialdehyde and Glutathione Assays

Brain tissue samples were homogenized in ice-cold 150 mM KCl for the determination of malondialdehyde and glutathione levels. The MDA levels were assayed for products of lipid peroxidation. Results were expressed as nanomole malondialdehyde gram^−1^ tissue. GSH was determined by the spectrophotometric method using Ellman’s reagent, and the results were expressed as micromole glutathione gram^−1^ tissue.

##### Luminol and Lucigenin Assays

Reactive oxygen species signals were made chemiluminescent by one of the following enhancer probes: Lucigenin (100 μM) or Luminol (1 mM). Brain tissues were thawed and washed with saline. Luminescence of the tissue samples was recorded at room temperature using a luminometer (Bad Wildbad, Germany) in the presence of enhancers. Tissue specimens were placed into tubes containing PBS-HEPES buffer (0.5 mol/L phosphate buffered saline containing 20 mmol/L HEPES, pH 7.2). ROS signals were quantitated after addition of the enhancer (lucigenin or luminol) to a final concentration of 0.2 mmol/L. After the measurements, the tissues were dried on filter papers and weighed. All chemiluminometric counts were obtained at 1-min intervals for 5 min, and the results were expressed as relative light units (Rlu) for 5 min per milligram of tissue.

##### Statistics

Each of the conditions in the study consists of data independent from all the others. Statistical comparisons have been made by use of one-way analysis of variance and Dunnet’s multiple comparisons test as well as Student’s *t* test of independent samples. As randomization has been performed only within each of the three sets of experiments, statistical analysis can only be made for each set independently from the others.

## Results

**Fig. 1:**
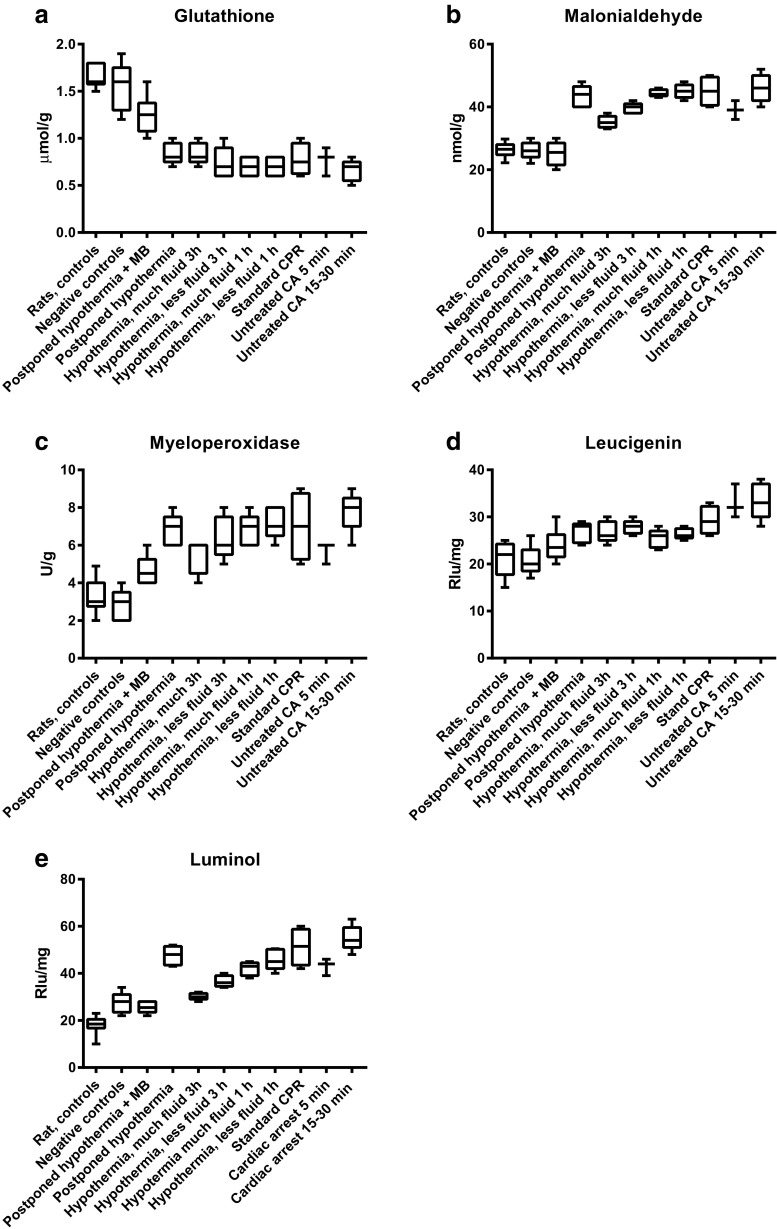
Indicators (**a** glutathione; **b** malonialdehyde; **c** myeloperoxidase; **d** leucigenin; **e** luminol) of tissue oxidative injury in different groups of piglets. Block diagrams indicating mean value and first and third quartiles as well as range

In the first set of the experiments (Fig. 1), the rat laboratory internal controls and untreated cardiac arrests (negative controls) should be regarded as a descriptive part of the present study as especially the animals subjected to longer untreated cardiac arrest are few. In spite of this limitation, it seems clear that the results from the negative controls are in reasonable agreement with the rat internal controls of the laboratory. Furthermore, a substantial part of the oxidative injury occurs early after cardiac arrest. Thus, there was a difference only for luminol (*P* = 0.02) between 5-min untreated cardiac arrest and those after 15–30-min untreated cardiac arrest.

**Fig. 2:**
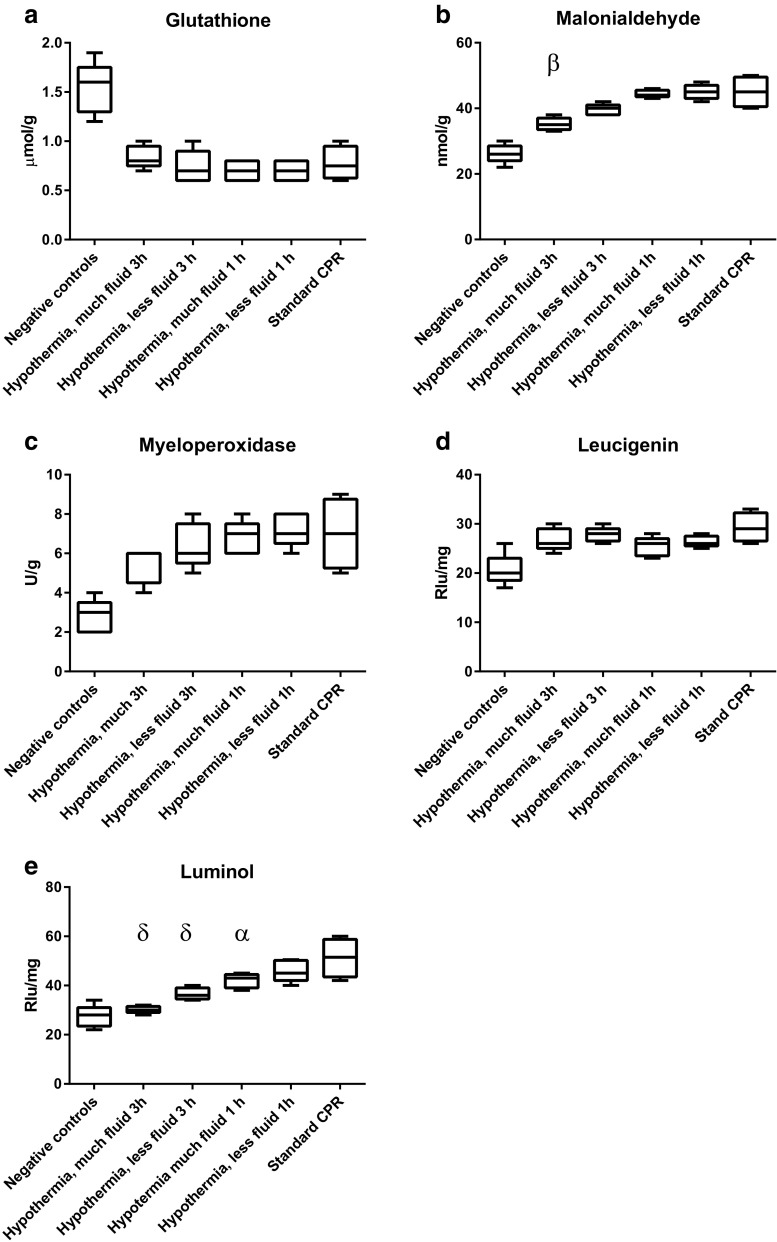
Indicators (**a** glutathione; **b** malonialdehyde; **c** myeloperoxidase; **d** leucigenin; **e** luminol) of tissue oxidative injury in second set of experiments. Block diagrams indicating mean value and first and third quartiles as well as range. Statistical differences (Dunnet’s multiple comparisons test) denoted above blocks refer to comparisons with *Standard CPR*: *P* < 0.05 as α; *P* < 0.01 as β; *P* < =0.001 as γ; and *P* < 0.0001 as δ. Negative controls not included in statistical analysis

In the second set of experiments (Fig. 2) where mild hypothermia was induced immediately after ROSC by external cooling combined with varying amounts of cold intravenous fluid, there was a difference (0.0001 < *P* < 0.001) between standard CPR and cooling with much or less fluid when estimated 3 h after ROSC by malonialdehyde and luminol. The other indicators of oxidative injury did not show a significant difference to standard CPR. In addition, we found that inducing mild hypothermia immediately after ROSC with less fluid exhibited significantly (0.01 < *P* < 0.05) less oxidative injury as indicated by luminol but not by the other indicators when estimated 1 and 3 h after ROSC. The effect on oxidative indicators luminol and malonialdehyde was greater 3 h as compared with 1 h after ROSC (0.0001 < *P* < 0.01).

**Fig. 3:**
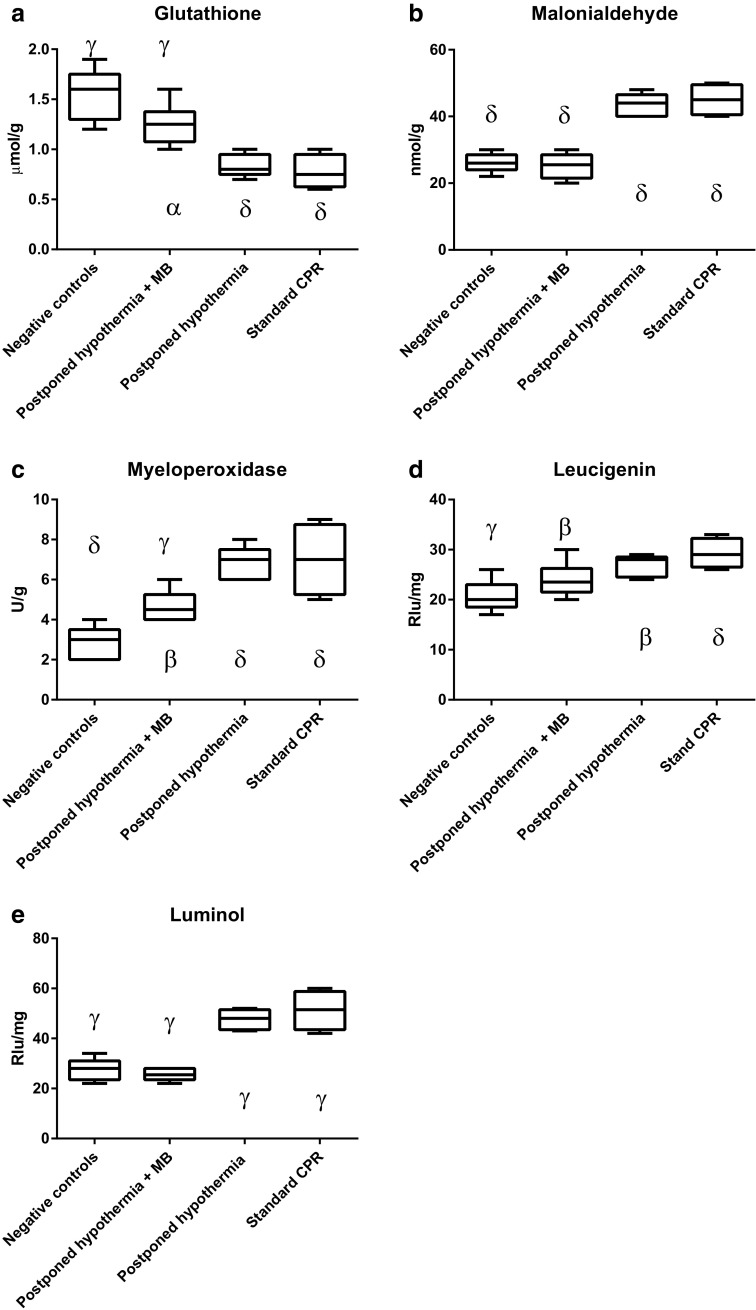
Indicators (**a** glutathione; **b** malonialdehyde; **c** myeloperoxidase; **d** leucigenin; **e** luminol) of tissue oxidative injury in third set of experiments. Block diagrams indicating mean value and first and third quartiles as well as range. Statistical differences (Dunnet’s multiple comparisons test) denoted above blocks refer to comparisons with *Standard CPR* and those denoted below blocks refer to comparisons with *Negative controls* as: *P* < 0.05 as α; *P* < 0.01 as β; *P* < =0.001 as γ; and *P* < 0.0001 as δ

In the third set of experiments (Fig. 3) comparing postponed hypothermia with and without addition of MB with standard CPR as well as negative controls, there was a significant difference between standard CPR and postponed hypothermia plus MB in all the indicators of oxidative injury (0.0001 < *P* < 0.05). Postponed hypothermia and standard CPR did not differ for any of the indicators of oxidative injury. When postponed hypothermia with MB was compared with negative controls, we found no statistical difference in any of the analyzed indicators of oxidative injury except for glutathione and myeloperoxidase. In contrast, there was a difference (0.0001 < *P* < 0.01) between negative controls and postponed hypothermia in all of the indicators of oxidative injury. There was also a significant difference between postponed hypothermia and postponed hypothermia with MB (0.0001 < *P* < 0.05) in all indicators of oxidative injury but leucigenin.

## Discussion

It seems important to point out that the present results were obtained from three different sets of studies: The first set involves data recorded from control animals (untreated CA); second set, animals subjected to CA, CPR, and immediately induced mild hypothermia as well as a group subjected to standard CPR; and third set, animals subjected to CA, CPR, and postponed hypothermia with and without administration of MB, compared to groups subjected to standard CPR as well as negative control animals (anesthetized animals, no CA, no CPR). Comparisons can be made inside these three sets but not between the three sets of experiments simply because randomization only was done within each set. However, all three sets of experiments were performed in the same lab with the same staff and attendant leadership within a narrow time frame. Due to these circumstances, differences between the three sets of experiments may serve as descriptive indicators of the extent of oxidative injury inflicted to the cerebral tissue during CA and after CPR (Fig. 1). The most important findings from the control animals are the serious oxidative cerebral injury that results from 5-min untreated CA that seems not to worsen much if the untreated cardiac arrest is allowed to continue for 15–30 min, maybe because the injury was near maximal already at 5 min. Sadly, treatment by “standard CPR” seems to result in more or less the same cerebral oxidative injury, but with an increased interquartile range, probably reflecting the biological variation of animals and varying success of the resuscitative efforts. In contrast to the major cerebral oxidative injury, early during the cardiac arrest are the microscopic findings of neuronal injury that evolve continuously during a much longer period during the reperfusion [1].

Induction of mild hypothermia has been a frequently used clinical method after its development [13–16]. The present results in the second set (Fig. 2) of experiments show a small but significant effect especially when large volume of cold saline is used for the rapid induction of hypothermia immediately after ROSC. Also, this is in agreement with our previous study where neuronal injury was studied by neuropathological methods [11].

These findings are in covenant with the results obtained in the third set (Fig. 3) of experiments showing a very modest effect on indicators of oxidative cerebral injury when the induction of mild hypothermia was postponed for 30 min, a finding that is in agreement with our own neuropathological findings as well as a large clinical controlled randomized trial [10, 17]. In contrast, a substantial positive effect was recorded when combining the postponed hypothermia with administration of MB during and after CPR [10]. This effect was great in spite of the weak effect of the postponed hypothermia, strengthening the previously recorded beneficial effects of MB on both survival and extent of cerebral neuropathological injury [1, 6]. In fact, administration of MB in combination with postponed cooling resulted in no statistical difference in the majority of the indicators of oxidative injury when this treatment was compared with the negative controls, i.e., animals just subjected to anesthesia. This finding also means that MB compensated for the oxidative injury that was recorded with postponed cooling. Our previously published neuropathological microscopic findings seem to agree with and confirm the present results.

### Limitations

Our determinations of cerebral tissue indicators of oxidative injury were performed on three sets of experiments, and randomization was only done inside each set, not between them. In spite of this limitation, we think that the results are performed within a laboratory with great experience in executing these kinds of repetitive experimental trials of cardiac arrest and cardiopulmonary resuscitation, and the results seem largely be in line with many clinicians’ experience as well as our own previously published experimental neuropathological results.

## Conclusions

An experimental cardiac arrest of long duration results in significant alteration of cerebral oxidative injury as indicated by analyses of malonialdehyde, myeloperoxidase, leucigenin, luminol, and glutathione indicating great harm that does not increase greatly after 5-min untreated cardiac arrest. The oxidative injury that results after successful cardiopulmonary resuscitation is significantly reduced by administration of methylene blue during and after CPR, followed by mild hypothermia induced 30 min after reestablishment of spontaneous circulation. Thus, while the effect of induced mild hypothermia in all the present experiments seems to be modest and deteriorates when induction of hypothermia is delayed, addition of MB is effective enough to compensate for 30-min postponement of hypothermia.

## References

[1] Sharma HS, Miclescu A, Wiklund L (2011). Cardiac arrest-induced regional blood-brain barrier breakdown, edema formation and brain pathology: a light and electron microscopic study on a new model for neurodegeneration and neuroprotection in porcine brain. J Neural Transm.

[2] Miclescu A, Sharma HS, Martijn C, Wiklund L (2010). Methylene blue protects the cortical blood-brain barrier against ischemia/reperfusion-induced disruptions. Crit Care Med.

[3] Mortberg E, Cumming P, Wiklund L, Rubertsson S (2009). Cerebral metabolic rate of oxygen (CMRO2) in pig brain determined by PET after resuscitation from cardiac arrest. Resuscitation.

[4] Basu S, Nozari A, Liu XL, Rubertsson S, Wiklund L (2000). Development of a novel biomarker of free radical damage in reperfusion injury after cardiac arrest. FEBS Lett.

[5] Basu S, Liu X, Nozari A, Rubertsson S, Miclescu A, Wiklund L (2003). Evidence for time-dependent maximum increase of free radical damage and eicosanoid formation in the brain as related to duration of cardiac arrest and cardio-pulmonary resuscitation. Free Radic Res.

[6] Miclescu A, Basu S, Wiklund L (2006). Methylene blue added to a hypertonic-hyperoncotic solution increases short-term survival in experimental cardiac arrest. Crit Care Med.

[7] Liu XL, Wiklund L, Nozari A, Rubertsson S, Basu S (2003). Differences in cerebral reperfusion and oxidative injury after cardiac arrest in pigs. Acta Anaesthesiol Scand.

[8] Wiklund L, Basu S, Miclescu A, Wiklund P, Ronquist G, Sharma HS (2007). Neuro- and cardioprotective effects of blockade of nitric oxide action by administration of methylene blue. Ann N Y Acad Sci.

[9] Idris AH, Roberts LJ, Caruso L, Showstark M, Layon AJ, Becker LB, Vanden Hoek T, Gabrielli A (2005). Oxidant injury occurs rapidly after cardiac arrest, cardiopulmonary resuscitation, and reperfusion. Crit Care Med.

[10] Wiklund L, Zoerner F, Semenas E, Miclescu A, Basu S, Sharma HS (2013). Improved neuroprotective effect of methylene blue with hypothermia after porcine cardiac arrest. Acta Anaesthesiol Scand.

[11] Miclescu A, Sharma HS, Wiklund L (2013). Crystalloid vs. hypertonic crystalloid-colloid solutions for induction of mild therapeutic hypothermia after experimental cardiac arrest. Resuscitation.

[12] Pulli B, Ali M, Forghani R, Schob S, Hsieh KL, Wojtkiewicz G, Linnoila JJ, Chen JW (2013). Measuring myeloperoxidase activity in biological samples. PLoS One.

[13] Bernard SA, Buist M (2003). Induced hypothermia in critical care medicine: a review. Crit Care Med.

[14] Bernard SA, Gray TW, Buist MD, Jones BM, Silvester W, Gutteridge G, Smith K (2002). Treatment of comatose survivors of out-of-hospital cardiac arrest with induced hypothermia. N Engl J Med.

[15] Sterz F, Safar P, Tisherman S, Radovsky A, Kuboyama K, Oku K (1991). Mild hypothermic cardiopulmonary resuscitation improves outcome after prolonged cardiac arrest in dogs. Crit Care Med.

[16] Walters JH, Morley PT, Nolan JP (2011). The role of hypothermia in post-cardiac arrest patients with return of spontaneous circulation: a systematic review. Resuscitation.

[17] Nielsen N, Wetterslev J, Cronberg T, Erlinge D, Gasche Y, Hassager C, Horn J, Hovdenes J (2013). Targeted temperature management at 33 degrees C versus 36 degrees C after cardiac arrest. N Engl J Med.

